# Dentistry and Travel Medicine – What is Keeping Them Apart?

**DOI:** 10.1016/j.identj.2025.03.014

**Published:** 2025-04-04

**Authors:** Irmgard L. Bauer

**Affiliations:** College of Healthcare Sciences, Academy—Tropical Health and Medicine, James Cook University, Townsville QLD, Australia

To the Editor

January to September 2024 recorded 1.1 billion international arrivals (overnight visitors).^1^ A great many of them will have consulted a travel health professional in preparation for a safe trip. For over 25 years, the specialty of travel medicine, represented by the International Society of Travel Medicine, the Faculty of Travel Medicine, Royal College of Physicians and Surgeons (Glasgow) (FTM RCPSG), and regional and national travel medicine societies, provides pretravel care, advice, vaccinations, and consideration of pre-existing medical conditions as well as assistance during travel and post-travel care. Travel health professionals are doctors, nurses, and pharmacists with specialist travel medicine qualifications serving in travel clinics, doctors’ surgeries, and pharmacies.

Peculiarly, travel medicine attends to all a traveller's body parts but one – the mouth. It is highly unlikely that people, even those attending regular dental checks, visit a dentist just for some tips for an upcoming trip. Like any other part of the body, while away from home, teeth can be a bother from a mild toothache to life-threatening emergencies, yet nobody seems to oversee advising travellers^2^ regarding preparation, potential problems or what to do during an emergency. In addition, routine dental hygiene is often curtailed during travel, made worse by an abundance of cariogenic food due to a lack of healthier options. A closer exploration of practical aspects of dental care during travel uncovers a number of unexpected problems.^3^ For example, the impact of toilet-plume-contaminated toothbrushes, especially when travelling with a travel companion or cabin mate who happens to have traveller's diarrhoea (*Escherichia coli*, norovirus, etc.), has never been investigated.

Unfortunately, the historical division between dentistry and medicine still prevails. Travel health professionals are no dentists but there is a fantastic opportunity for both specialties to come together and bridge this unproductive division by collaborating on innumerable research topics and preparing evidence-based guidelines for dental travel health so that travellers can travel with all their body parts looked after.^3^

## Conflict of interest

The authors declare that they have no known competing financial interests or personal relationships that could have appeared to influence the work reported in this article.
